# Nucleoside Analog Reverse-Transcriptase Inhibitors in Membrane Environment: Molecular Dynamics Simulations

**DOI:** 10.3390/molecules28176273

**Published:** 2023-08-27

**Authors:** Anna Stachowicz-Kuśnierz, Beata Korchowiec, Jacek Korchowiec

**Affiliations:** Faculty of Chemistry, Jagiellonian University, Gronostajowa 2, 30-387 Krakow, Poland; anna2.stachowicz@uj.edu.pl (A.S.-K.); bkorch@chemia.uj.edu.pl (B.K.)

**Keywords:** phospholipid membranes, nucleoside reverse-transcriptase inhibitors, ddC, ddI, d4T, ATC, molecular dynamics simulations, monolayer/bilayer model membranes

## Abstract

The behavior of four drugs from the family of nucleoside analog reverse-transcriptase inhibitors (zalcitabine, stavudine, didanosine, and apricitabine) in a membrane environment was traced using molecular dynamics simulations. The simulation models included bilayers and monolayers composed of POPC and POPG phospholipids. It was demonstrated that the drugs have a higher affinity towards POPG membranes than POPC membranes due to attractive long-range electrostatic interactions. The results obtained for monolayers were consistent with those obtained for bilayers. The drugs accumulated in the phospholipid polar headgroup region. Two adsorption modes were distinguished. They differed in the degree of penetration of the hydrophilic headgroup region. Hydrogen bonds between drug molecules and phospholipid heads were responsible for adsorption. It was shown that apricitabine penetrated the hydrophilic part of the POPC and POPG membranes more effectively than the other drugs. Van der Waals interactions between S atoms and lipids were responsible for this.

## 1. Introduction

Nucleoside analog reverse-transcriptase inhibitors (NRTIs) are synthetic antiretroviral drugs used in the treatment of human immunodeficiency virus (HIV) infection and acquired immunodeficiency syndrome (AIDS) [1]. Together with non-nucleoside reverse-transcriptase inhibitors [2] and protease inhibitors [3], they lay at the heart of highly active antiretroviral therapy (ART) [4,5]. ART has been extremely successful in reducing morbidity and mortality caused by HIV/AIDS. The development of ART enabled turning HIV/AIDS into a chronic yet manageable illness and greatly extended the lifespan of HIV-infected individuals [6,7]. ART is also used in both pre- and postexposure prophylactic treatment strategies in order to limit the spread of HIV [8,9,10].

The antiviral action of ART is designed to block HIV replication at various stages of the virus’ life cycle. In the case of NRTIs, the mechanism firstly proceeds via intracellular phosphorylation by deoxycytidine kinase to the active triphosphate forms [11]. After activation, NRTIs compete with endogenous triphosphates for binding to viral reverse transcriptase and subsequent incorporation into DNA strands [12,13,14]. Since NRTIs lack the 3′-hydroxyl group, such an event results in chain termination and, finally, inhibition of reverse transcription. Non-nucleoside reverse-transcriptase inhibitors block the enzyme allosterically by inducing conformational changes [15,16]. In turn, HIV protease inhibitors prevent maturation of viral proteins [3]. Finally, entry inhibitors preclude insertion of the viral material into host cells [17,18]. The combined effect of these drugs allows for the suppression of plasma levels of HIV RNA (viral load) below the detectable limit.

Despite this unquestionable success of ART, some issues still require addressing. The most serious of them are connected with infection rebound if ART is interrupted or discontinued. Since ART only suppresses HIV infection, insufficient treatment adherence results in restored high HIV levels, increased morbidity, and resumption of potency to transmit the infection. It may also lead to proliferation of drug-resistant mutants [19]. Infection rebound is connected with the existence of infection reservoirs and sanctuary sites, i.e., cells containing replication-competent viral material, which are resistant to treatment [20,21,22]. It was shown that the development of these reservoirs is related to the fact that they reside in locations unreachable by the ART drugs, e.g., lymphoid tissue [23,24,25], the central nervous system [26,27], or the genital tract [28,29]. In order to penetrate these regions, the drugs first have to pass physiological barriers, such as the blood–brain, blood–cerebrospinal fluid, or blood–testis barrier. Commonly accepted mechanisms for transport of NRTIs across these barriers include passive diffusion and active transport with the use of membrane transporters [30,31]. It is therefore of interest to study interactions between NRTIs and phospholipid membranes in order to allow improvement in their uptake.

**Scheme 1:**
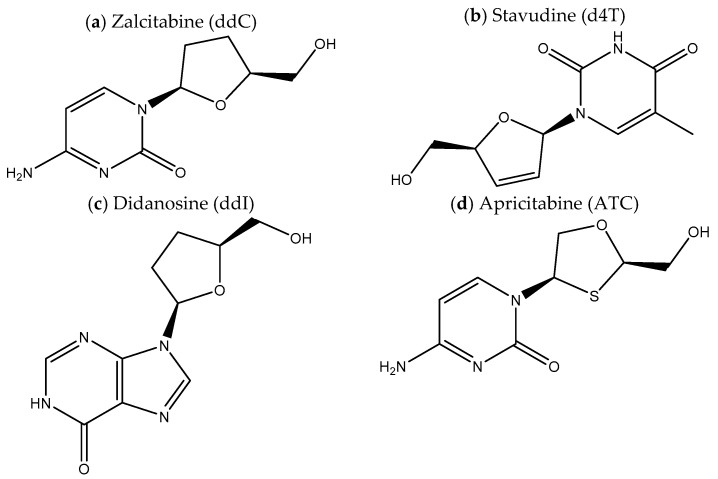
Chemical structures of the four NRTIs used in this work: zalcitabine, ddC (**a**), stavudine, d4T (**b**), didanosine, ddI (**c**), and apricitabine, ATC (**d**).

In this study, four NRTI drug molecules were examined, namely zalcitabine (ddC) [32,33], stavudine (d4T) [34], didanosine (ddI) [35], and apricitabine (ATC) [36]. Their chemical structures are shown in Scheme 1. ddC, ddI, and d4T are examples of first-generation NRTIs, approved for use in the early 1990s [37]. ATC was discovered more recently, in response to adverse effects caused by the NRTIs of the previous generation [38] and resistance issues [39,40]. We have employed molecular dynamics (MD) simulations to study the behavior of NRTIs in model lipid monolayers and bilayers. In the past decades, MD simulations have become a valuable tool in studies of biological systems. They give insight into atomistic/molecular details which are unavailable to direct experimental measurements. In some of our previous works, we have demonstrated that MD simulations can be helpful in studying drug–membrane interactions [41,42]. They were also successfully used in studies of the interactions between NRTIs and reverse transcriptase [43,44,45,46,47]. The aim of this study was to examine mutual interactions between the selected NRTIs and phospholipid membranes. One-component POPC or POPG systems were considered. POPC is commonly used as a universal model lipid to study biological membranes. POPG was chosen to examine the influence of headgroup charge on the behavior of NRTIs. Clearly, such simple, one-component systems do not capture the complexity of biological membranes. However, we were more concerned with elucidating clear trends and thus decided to keep the models as simple as possible. We analyze the drugs’ adsorption to the membranes and rationalize the observed trends in terms of their electronic structure on the grounds of conceptual density functional theory. The energetics of NRTI–lipid interactions is also analyzed with semiempirical methods. The membranes were constructed in two forms: monolayers and bilayers. The behavior of both membrane types is compared to check whether in this case a monolayer system can be regarded as a good model of a bilayer. The former has some advantages in terms of experimental studies of drug–membrane interactions. Most importantly, in monolayer systems, surface area per lipid can be easily controlled.

## 2. Results and Discussion

### 2.1. Molecular Dynamics Simulations

**Figure 1:**
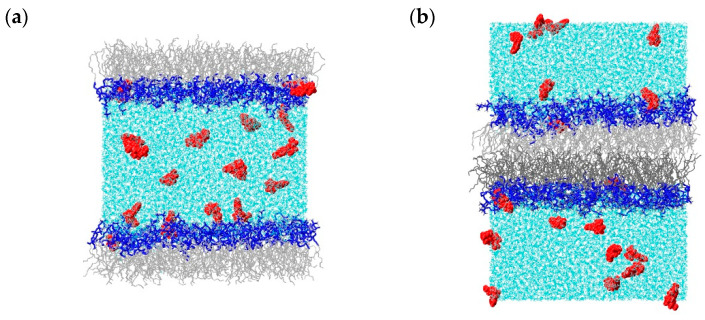

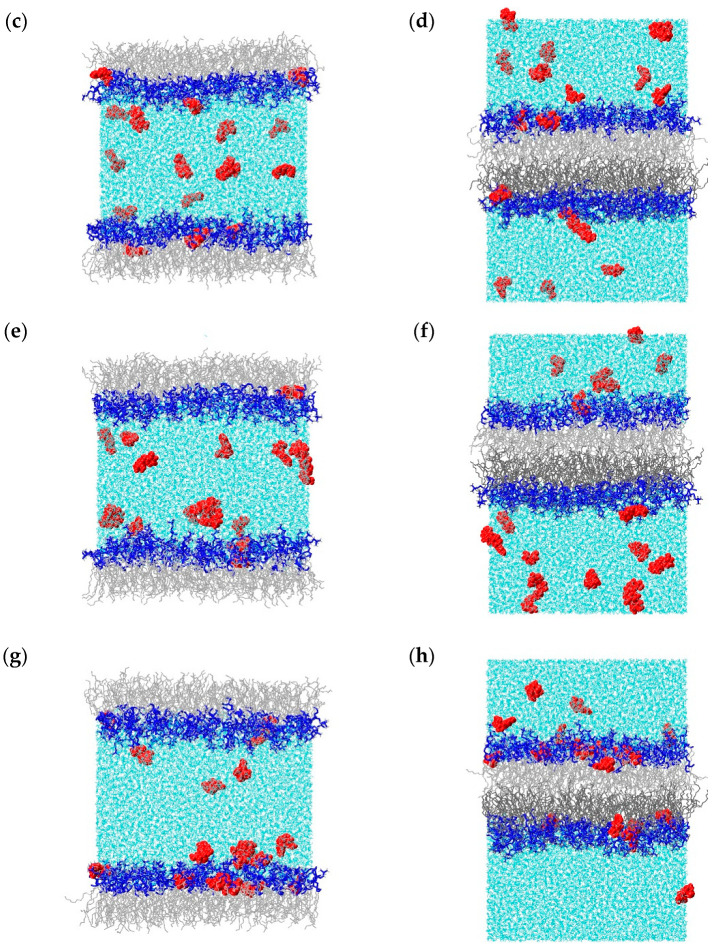
POPC monolayer (left column, panels (**a**,**c**,**e**,**g**)) and bilayer (right column, panels (**b**,**d**,**f**,**h**)) with zalcitabine (ddC, first row, (**a**,**b**)), stavudine (d4T, second row, (**c**,**d**)), didanosine (ddI, third row, (**e**,**f**)), and apricitabine (ATC, fourth row, (**g**,**h**)). Color code: red—drug molecules, blue—POPC headgroups, gray—POPC hydrophobic tails, cyan—water.

**Figure 2:**
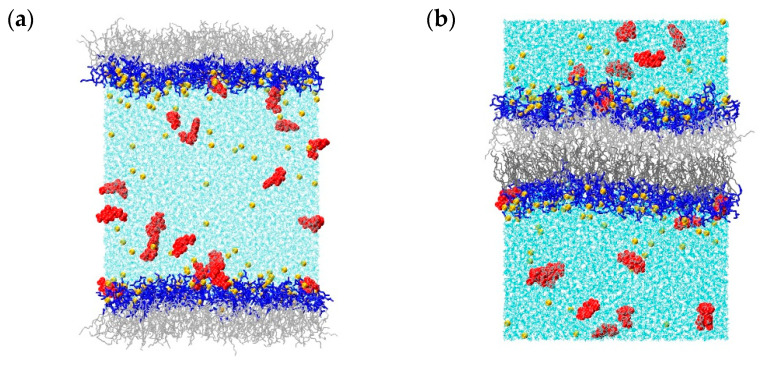

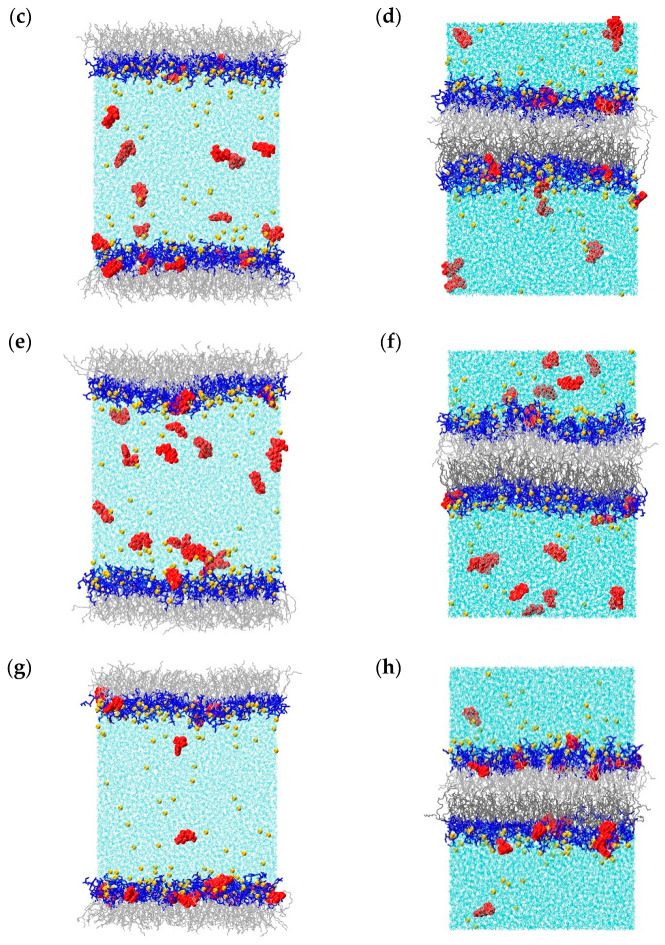
POPG monolayer (left column, panels (**a**,**c**,**e**,**g**)) and bilayer (right column, panels (**b**,**d**,**f**,**h**)) with zalcitabine (ddC, first row, (**a**,**b**)), stavudine (d4T, second row, (**c**,**d**)), didanosine (ddI, third row, (**e**,**f**)), and apricitabine (ATC, fourth row, (**g**,**h**)). Color code: red—drug molecules, blue—POPG headgroups, gray and black—POPG hydrophobic tails, cyan—water, yellow—Na^+^.

Side views of all molecular systems examined here are shown in Figure 1 and Figure 2. The left and right columns in both figures correspond to monolayer and bilayer systems, respectively. POPC monolayers/bilayers are collected in Figure 1, while POPG systems are shown in Figure 2. The drug molecules are marked as red balls. The subsequent rows correspond to different antiretroviral drugs: zalcitabine (ddC), stavudine (d4T), didanosine (ddI), and apricitabine (ATC). Visual inspection of the obtained structures already leads to several qualitative conclusions. Firstly, the behavior of drug molecules was similar in monolayer and bilayer systems. Secondly, all of them have higher affinities for anionic POPG than for zwitterionic POPC lipids. Among the four examined drugs, ATC shows the greatest affinity for phospholipid membranes. Strong adsorption of ATC molecules to the hydrophilic parts of the POPC and POPG monolayers and bilayers can be observed. d4T also readily adsorbs to the hydrophilic parts of the POPG membranes, but its affinity to POPC is smaller than that of ATC. The remaining molecules adsorb to the monolayer/bilayer surface, but penetration into the hydrophilic parts of the membranes is limited. Penetration deep into the hydrophobic parts of the membranes was not observed. This can be easily understood since these drugs are well soluble in water and their water/octanol partition coefficients are negative.

**Figure 3:**
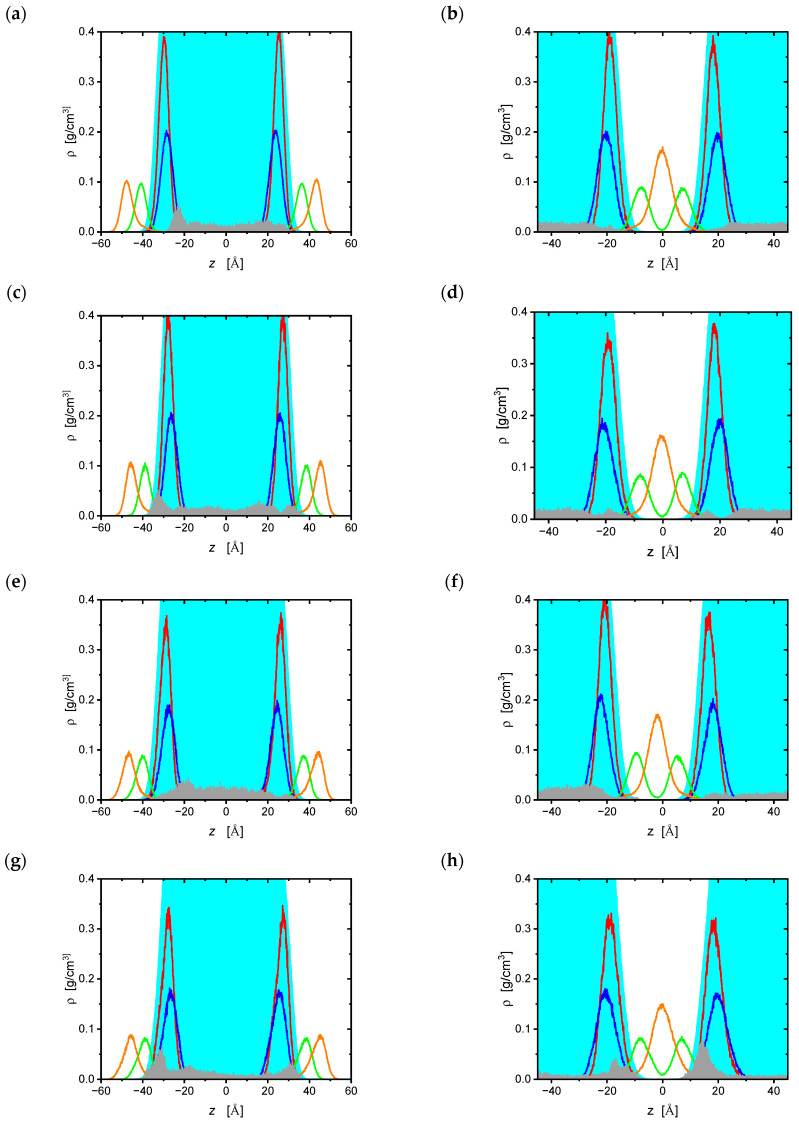
Partial density plots, ρ=ρ(z), for POPC monolayers (left column) and bilayers (right column): zalcitabine (**a**,**b**), stavudine (**c**,**d**), didanosine (**e**,**f**), and apricitabine (**g**,**h**). Color code: cyan—water, red lines—PO_4_ groups, blue—N(CH_3_)_3_, green—C=C from *sn*-2 chains, orange—terminal CH_3_ groups of hydrophobic tails, gray area—center of mass of drug molecules.

**Figure 4:**
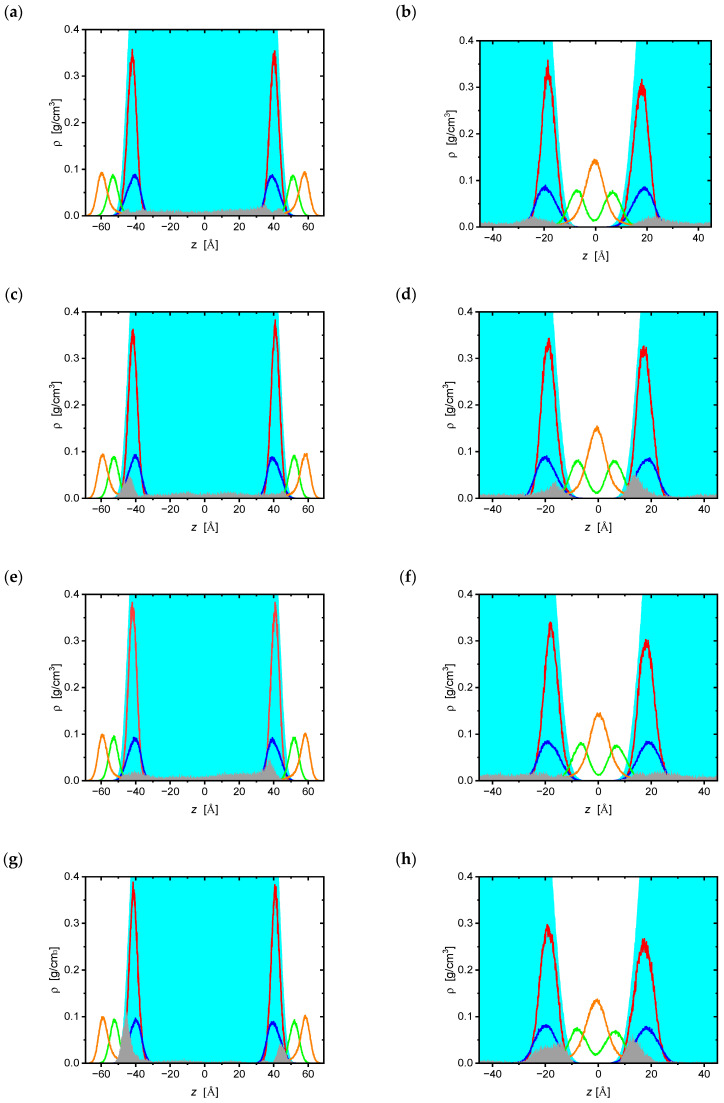
Partial density plots, ρ=ρ(z), for POPG monolayers (left column) and bilayers (right column): zalcitabine (**a**,**b**), stavudine (**c**,**d**), didanosine (**e**,**f**), and apricitabine (**g**,**h**). Color code: cyan—water, red lines—PO_4_ groups, blue—OH of POPG, green—C=C from *sn*-2 chains, orange—terminal CH_3_ groups of hydrophobic tails, gray area—center of mass of drug molecules.

The above qualitative observations are confirmed when partial density plots for various system components are analyzed. They are shown in Figure 3 and Figure 4. The arrangement of panels in these figures is the same as in Figure 1 and Figure 2. The same functional groups were plotted for mono- and bilayer systems. They include hydrophilic PO_4_ and choline (POPC) or hydroxyl (POPG) functional groups (red and blue colors, respectively), *sn*-2 double bonds (green), and terminal CH_3_ groups of the hydrophobic tails (orange). Water is marked with cyan areas. The positions of the drug molecules’ center of mass are shown as gray areas.

As far as lipid components are concerned, it can be seen that the partial density plots are almost symmetric. This is not surprising since phospholipids only diffuse laterally. The vertical component of diffusion may appear for high surface pressures when membrane collapse occurs. On the other hand, the partial density plots of the drug molecules are not symmetric, which is due to asymmetric distribution between the two monolayers present in the systems. In bilayer systems, drug molecules diffuse across periodic cell walls. Such a situation does not appear in monolayer systems due to the presence of the vacuum slab; crossing the water–vacuum interface is associated with a large energy cost (hydration energy). Considering the asymmetric distribution of the drug molecules, their interactions with the upper/lower monolayer or the upper/lower bilayer leaflet can be regarded as independent simulations.

Accumulation of drug molecules in the headgroups region is manifested by distinct maxima in ρ(z) plots for most drugs. This is connected with adsorption to phospholipid headgroups. Two adsorption modes can be distinguished. They differ in the degree of penetration into the lipid heads: (*i*) adsorption to the monolayer/bilayer surface and (*ii*) adsorption into the headgroup region. In density plots, these two modes manifest as distinct maxima along the *z*-axis. Those corresponding to adsorption into the headgroups are clearly shifted towards the interface. The behavior of the drug molecules in monolayer and bilayer systems is consistent. The distribution of NRTIs across the water slab was uniform, i.e., no clustering of the drug molecules in solution was observed.

The higher affinity of the examined NRTIs towards anionic POPG than towards the zwitterionic POPC is evidenced by the more pronounced maxima in the ρ(z) plots. Comparison between the drugs shows that ATC stands out from the remaining molecules in terms of its large affinity towards the membranes. For the other NRTIs, the tendency to accumulate in the membranes is smaller and diminishes in the following series: d4T > ddI/ddC.

There is a small difference between POPC and POPG bilayers in terms of their thickness. Namely, the POPC bilayer, which is slightly more ordered than that of POPG, is thicker. Chain interpenetration in both systems is similar, but in POPC the thickness of the hydrophobic part is greater. This is manifested in the shape and position of the partial density plots of the terminal CH_3_ groups. For POPG bilayers, these plots are lower and wider than for POPC bilayers (the same normalization is maintained). This can be rationalized in terms of repulsion between the anionic headgroups of PG, which results in a higher area per lipid (APL). When pure POPG and POPC bilayers were equilibrated in an *NpT* ensemble (310 K, 1 atm), the APL stabilized at 69.4 and 67.3 Å^2^/lipid, respectively. Structural parameters of the bilayer and monolayer systems are summarized in Table 1. Bilayer thickness was defined as the average distance between the phosphorus atoms of opposite leaflets. The thickness of the hydrophobic part was defined as the average distance between the C2 carbon atoms of opposite leaflets. In the case of the monolayer systems, terminal carbon atoms were used instead of other leaflet P/C2 atoms. In all systems, the thickness of the POPC/POPG bilayer is more than twice the thickness of the monolayer. This indicates greater disorder in the hydrophobic chains in monolayer systems. The presence of drug molecules does not change the structural parameters.

**Figure 5:**
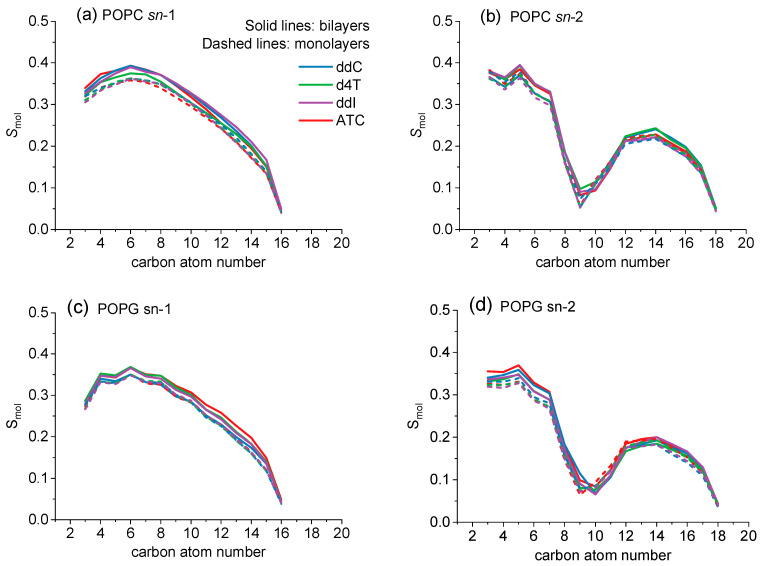
Comparison of lipid chain order parameters in bilayer (solid lines) and monolayer (dashed lines) systems with the four studied NRTIs. (**a**) *sn*-1 chains in POPC, (**b**) *sn*-2 chains in POPC, (**c**) *sn*-1 chains in POPG, (**d**) *sn*-2 chains in POPG.

The effect of drug molecules on lipid chains is summarized in Figure 5, where chain order parameters (*S*_mol_) in *sn*-1 and *sn*-2 chains are plotted. In the case of POPC, the curves are overlapping and only a small broadening is observed. *S*_mol_ values in the bilayers (solid lines) are higher than in the monolayers (dashed lines). This shows that bilayers are slightly more ordered than monolayers at the same surface area. The same relationship is observed in POPG systems. The order in the POPC bilayer/monolayers is slightly higher than in the POPG systems. A slight ordering effect of ATC molecules in the POPG monolayer can also be observed. This is manifested by increased *S*_mol_ values obtained with this drug.

**Figure 6:**
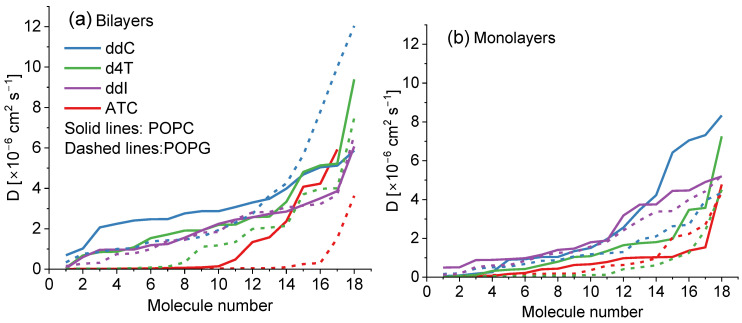
Diffusion coefficients of drug molecules in bilayer (panel (**a**)) and monolayer (panel (**b**)) systems. The data were obtained from trajectories of 60 ns.

Diffusion coefficients (*D*) are good descriptors to analyze the behavior of drug molecules. We have calculated their values from the second half of the simulations (60 ns) according to the Enstein relationship [48]. The obtained coefficients, sorted in ascending order, are shown in Figure 6. It can be seen that the *D* values fall into two ranges: small values below c.a. 2 × 10^−6^ cm^2^ s^−1^ and larger values. Inspection of the trajectories reveals that molecules with small *D* values are the ones penetrating into the hydrophilic part of the membrane. The largest *D* values correspond to drug molecules in solution. Analysis of the plots shown in Figure 6 confirms the observations from the density profiles. Namely, ATC has the strongest tendency to accumulate in the membranes, especially in the POPG bilayer. The highest mobility in both systems is observed for the ddC molecule, indicating weak adsorption of this drug to the membranes. This large difference between ATC and ddC molecules suggests that interactions with ribose rings are important in terms of NRTI adsorption (ATC and ddC share the same cytidine base; see Scheme 1).

All examined NRTIs have a hydrophilic character. As was observed in Figure 1 and Figure 2, they either accumulated in the hydrophilic part of the monolayer/bilayer or stayed in the water. It can be expected that hydrogen bonds (HBs) are a strong stabilizing factor for adsorption of these molecules to the membranes. In Table 2, we summarize the average total number of HBs between drug molecules and phospholipid or water molecules. It was taken into account that NRTIs can be both donors and acceptors of hydrogen. A standard geometric criterion (donor–acceptor distance below 3 Å and angle cutoff of 20°) was used to count hydrogen bonds. The numbers collected in the table show that NRTIs interact more strongly with POPG than POPC. In almost every system, the average number of HBs between the drug and POPG is higher than in the case of POPC; the only exception is ddC in the monolayer model. Among the NRTIs, the highest number of HBs was counted for ATC, followed by d4T. This observation is consistent with the monolayer/bilayer side views and partial density plots. In both lipid environments, the drug molecules act as hydrogen donors. In the case of POPC, this mode is dominant and the acceptor contribution is negligible. The acceptor contribution slightly increases for POPG bilayers/monolayers; however, donor contribution remains more pronounced. These numbers correspond to 18 drug molecules. Another interesting relationship is the number of hydrogen bonds formed with water molecules, which in all cases is greater than 17. This can be explained by the strong hydration of the hydrophilic region of the membranes (see partial density plots in Figure 3 and Figure 4). In HBs with water, drug molecules again may act as hydrogen donors and acceptors. In this case, the acceptor contributions are dominant.

The common features of all the drug molecules are hydroxyl groups in the sugar ring, quinone oxygen atoms, and aromatic N/NH groups in the nucleobases. In ATC and ddC, amine NH_2_ groups are also present. All these moieties can form hydrogen bonds with lipids. Detailed analysis of the HB-count output shows that the majority of HBs are formed with hydrogen-donating groups of NRTIs, i.e., NH, NH_2_, and OH groups. In both the POPC and POPG membranes, they are primarily formed with PO_4_ oxygen atoms. Only in the case of ATC, especially in POPG systems, are some HBs formed with lipid ester groups. On the other hand, hydrogen-accepting interactions are scarcer, and have shorter lifetimes. They are formed by aromatic N atoms and quinone oxygens. In POPG, they are formed with glycerol OH groups. In POPC systems, they occur between NRTIs and positively charged N(CH_3_)_3_ groups. The latter case is not a standard hydrogen bond configuration. However, interactions with N(CH_3_)_3_ have a similar abundance and lifetime, as do those with OH of POPG. Therefore, it can be concluded that they fall into the extended IUPAC definition of HB: a *hydrogen bond is an attractive interaction between a hydrogen atom from a molecule or a molecular fragment X–H in which X is more electronegative than H, and an atom or a group of atoms in the same or a different molecule, in which there is evidence of bond formation* [49].

One can expect that adsorbed drug molecules are additionally stabilized by van der Waals contacts. This interaction is especially important when ddC and ATC molecules are compared. From Scheme 1, it can be seen that they only differ in the presence of S atoms in the ATC sugar ring. However, they represent the opposite membrane binding patterns—ATC binds the most readily, ddC the least. In order to understand this observation, we calculated averaged van der Waals and electrostatic interaction energies between NRTIs and lipids and between NRTIs and water. The NamdEnergy tool of VMD was used for this purpose. A total of 100 frames were extracted from the final 20 ns of the simulations. For each frame, NRTIs were first divided into two groups, molecules adsorbed to the membrane and molecules in solution, on the basis of their distance from the bilayer center. Next, the electrostatic and van der Waals contributions to the potential energy were calculated and averaged in each group. The results obtained for the simulations with POPC and POPG bilayers are reported in Tables S1 and S2 of the Supplementary Materials. It can be seen that in the case of ATC molecules adsorbed to the membrane, the presence of S atoms indeed results in a large van der Waals contribution to the drug–lipid interaction energy. The value obtained for this molecule is the largest among all the NRTIs. Interestingly, the electrostatic contribution is also the largest for this molecule. On the other hand, when molecules in solution are examined, both the electrostatic and van der Waals interaction energies of ATC and ddC with water are of similar magnitudes. It can be concluded that van der Waals interactions between S atoms of ATC and lipids are responsible for the increased tendency of this drug to accumulate in the POPC bilayer. The results obtained for POPG lead to similar conclusions. One more observation comes from the values reported in Tables S1 and S2. Namely, in the case of POPC systems, interaction energies between NRTIs and the membrane are negligible for molecules that are not adsorbed. On the other hand, in the case of the POPG systems, attractive electrostatic interaction between lipids and NRTIs was also detected for the molecules in solution. This explains the increased accumulation of the drug molecules in the POPG systems.

To complete this analysis, we calculated van der Waals and electrostatic contributions to the interaction energies between lipids and corresponding functional groups in the ATC and ddC molecules adsorbed to the POPC bilayer. These included the following: ribose ring O atoms, and the groups present in 3′ positions: S in ATC and CH_2_ in ddC. As expected, electrostatic interactions were dominant for O atoms. The average energy values were equal to −3.1 and −2.6 kcal/mol per molecule for ddC and ATC, respectively. Van der Waals stabilization was below 1 kcal/mol per molecule. However, for the S atoms, the van der Waals energy was −3.5 kcal/mol per molecule. It is therefore comparable to the energy of weak hydrogen bonds. For comparison, in ddC the van der Waals interaction between CH_2_ and lipids is equal to −1.8 kcal/mol per molecule.

### 2.2. Drug Donor/Acceptor Properties

**Figure 7:**
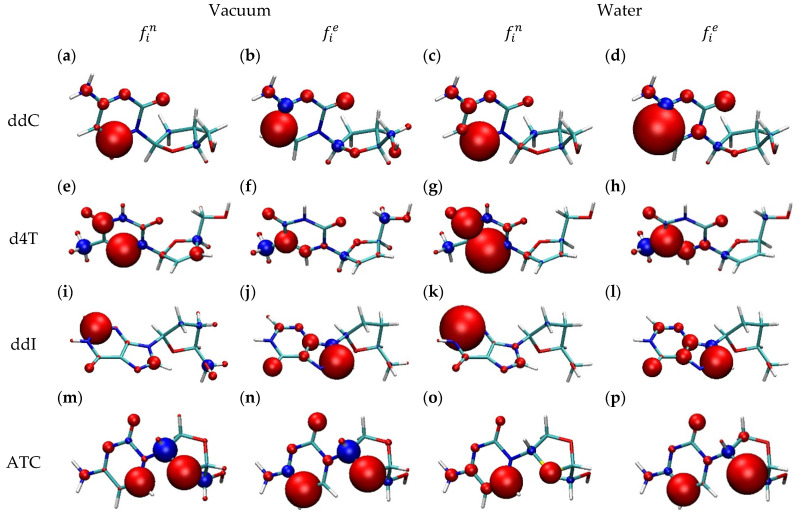
FF indices in vacuum (left half) and PCM water model (right half) for ddC (**a**–**d**), d4T (**e**–**h**), ddI (**i**–**l**), and ATC (**m**–**p**) molecules. The ball radii are proportional to absolute value of FF index. Red/blue balls corresponds to positive/negative values. Nucleophilic FF indices are in first and third columns while electrophilic are in second and fourth columns.

To further explore the observed differences between the NRTIs, especially the different behavior of ATC, we performed electronic structure calculations. Our previous works show that such calculations may provide valuable information in terms of drug–drug and drug–biomolecule interactions [50,51]. As shown in Scheme 1, the molecules analyzed here have several common structural features. Zalcitabine, apricitabine, and stavudine are derivatives of pyrimidine nucleosides. ddC and ATC are cytidine analogues, and d4T is an analogue of thymidine. Didanosine is an analogue of purine nucleoside, inosine. ddC and ddI share the same 2,3-dideoxyribose ring. In d4T, an unsaturated C2-C3 bond is introduced. Finally, in ATC, a 2-deoxy-3-oxa-4-tiorobose ring is present. We were interested in to what extent these similarities/differences would translate onto electronic structure and donor/acceptor properties. To check this, we performed electronic structure calculations at the B3LYP/6-311G(d,p) level of theory. To describe donor/acceptor properties, we calculated global and local (atomic resolution) charge sensitivities according to the formalism of conceptual density functional theory [52]. The results are summarized in Table 3 and Figure 7.

The conceptual density functional approach is well documented in the literature, so here only an overview is given as required. For more details, see for example the works of Geerlings et al. [52,53]. In short, this formalism approaches chemical reactivity in a way similar to traditional phenomenological thermodynamics. It is described in terms of its sensitivities (responses) to changes in the state parameters. The first (chemical potential, Equation (1)) and second (hardness, Equation (2)) derivatives of system energy (E) with respect to the number of electrons (N) in the system can be calculated with the finite difference method (parabolic fit):

(1) $$\mu ={\left(\frac{\partial E}{\partial N}\right)}_{v}≅-\frac{I+A}{2}$$

(2) $$\eta ={\left(\frac{\partial^{2}E}{\partial N^{2}}\right)}_{v}≅I-A$$

where A=E(N)−E(N+1) and I=E(N−1)−E(N) are electron affinity and ionization potential, respectively. All the energies E(N−1), E(N), and E(N+1)  were computed for the same external potential $v\left(\vec{r}\right)$, namely the fixed positions of all nuclei at the geometries of neutral molecules. The obtained values are collected in Table 3.

Electronic chemical potential (negative of electronegativity) is responsible for the direction of charge transfer. The values gathered in Table 3 show that the molecules analyzed here can be divided into two sets. The less electronegative ddC and ddI form one subgroup, while d4T and ATC are in the second subgroup. The effect of the environment on chemical potentials is rather small. The differences in both sets of data are smaller than 0.009 a.u. This is less than 7%. All molecules have the same global hardness, except for ATC, which is slightly softer. The soft sulfur atom in the five-membered ring is responsible for this effect. A strong environmental effect is observed for global hardnesses: in water, its values drop by more than 40% in comparison with its values in vacuum. It can be concluded that ATC and d4T have stronger acceptor properties than ddC and ddI. Therefore, one can expect stronger interaction with the anionic POPG monolayer/bilayer system. In zwitterionic POPC, ATC and d4T should have a stronger affinity towards PO_4_ units.

In Figure 7, we collect the Fukui function (FF) indices, being a measure of the local donor/acceptor properties of a molecule. We have previously shown that atomic FF indices can be used to probe electrophilic/nucleophilic properties on nucleobases [54]. Again, the finite difference approximation was used to approximate FF:

(3) $$f_{i}={\left(\frac{\partial N_{i}}{\partial N}\right)}_{v}=\{\begin{array}{c} f_{i}^{n}=N_{i}\left(N+1\right)-N_{i}\left(N\right)\\ f_{i}^{e}=N_{i}\left(N\right)-N_{i}\left(N-1\right)\\ f_{i}^{r}=\frac{1}{2}\left(f_{i}^{n}+f_{i}^{e}\right) \end{array}$$

where $N_{i}\left(N+1\right)$, $N_{i}\left(N\right)$, and $N_{i}\left(N-1\right)$ correspond to the electron populations of a given atom in an anion, neutral molecule, and cation, respectively. $f_{i}^{n},\;f_{i}^{e}$, and $f_{i}^{r}$ correspond to nucleophilic (*n*), electrophilic (*e*), and radical (*r*) reactions, respectively. Atomic charges fitted to molecular electrostatic potential (CHELPG scheme) were assumed to compute the FF indices.

It is clear from Figure 7 that in ddC, d4T, and ddI, the heterocyclic nucleobases are softer (large *f_i_* indices) than the five-membered rings (low *f_i_* indices). A slightly different picture is observed in the ATC molecule. The presence of a soft sulfur atom in the five-membered ring makes the ring softer. In this case, both parts of the molecule (sugar and nucleobase) can be regarded as active. This may explain increased membrane penetration via simultaneous favorable electrostatic and van der Waals interactions. This result is in line with the observations from MD simulations. The same observation is valid for nucleophilic and electrophilic FF distributions. The ring heteroatoms and substituted carbon atoms are harder than the other ring atoms. The majority of atoms in all panels work in phase (dominance of red balls). This is not surprising since FF indices are normalized to unity: $\underset{i}{\sum }f_{i}^{n}=1$ and $\underset{i}{\sum }f_{i}^{e}=1$. This means that a particular atom has the same properties as the whole molecule. Atoms with negative FF indices (blue balls) have opposite behavior to global ones. If a molecule acts as a donor/acceptor then these atoms have local acceptor/donor properties. In the ddI molecule, one five-membered ring is coupled with a six-membered one. Such coupling makes both rings relatively soft. In all systems, CH_2_OH groups are hard. Chinone oxygen, the nitrogen of the amino group, and the carbon of CH_3_ are harder than 1-3-diazine carbon atoms. In all systems, hydrogen atoms are very hard. Hard atoms are important in all interactions where the electrostatic contribution is dominant (hydrogen bonds), while soft atoms are important in those where polarization and charge-transfer contributions play a role (different van der Waals contacts). This explains the predominance of HBs formed with hydrogen-donating groups of NRTIs, i.e., the NH, NH_2_, and OH groups observed in the simulations. The FF picture does not change when going from the vacuum to the PCM model.

### 2.3. NRTI–Lipid Interaction Energies

In order to further analyze differences between the NRTIs’ affinities towards the membranes, we calculated interaction energies between the drugs and lipids. In Table 4, we present the energy change accompanying the adsorption process. Each number is the average of the interaction energy from about a hundred quantum-chemical calculations. The calculations were performed for systems cut from MD trajectories. Drug molecules together with all lipids with any atom within 3 Å from the NRTIs were cut. All calculations were performed using semiempirical AM1 Hamiltonian.

It is clear from Table 4 that ATC and d4T molecules have the greatest affinity to POPG monolayers and bilayers. In POPC systems, this dependence is somewhat distorted. The discrepancy occurs for ddI in the POPC monolayer: ddI has a higher affinity for POPC than d4T and ATC molecules. The results obtained for monolayers and bilayers are within the error bars. Adsorption to POPG is more favorable than to POPC. The differences between the energies are not large. In the POPC bilayer, they do not exceed 1.7 kcal/mol. In the POPG bilayer, the changes are slightly larger (2.4 kcal/mol). In the monolayer system, the differences are less pronounced.

## 3. Methods

Typical setup was used in construction of monolayer/bilayer systems [55]. Model POPC bilayer composed of 100 molecules in each leaflet was prepared using VMD programs, namely Membrane Builder, Add Solvation Box, and Add Ions modules. POPG monolayer was prepared with the Membrane Builder [56,57] functionality of CHARMM-GUI [58,59]. The bilayer oriented in the *xy* plane was put in a box of c.a. 20,000 water molecules. The initial size of water box was 87 × 87 × 120 Å. Both systems were subject to energy minimization and short equilibration in canonical ensemble. Next, 120 ns long simulations in the isothermal–isobaric ensemble were performed. C36 CHARMM force-fields for lipids [60] and TIP3P [61] water model were applied in all MD simulations. The temperature and pressure were set to 310 K and 1 bar, respectively. The ratio of box *x* and *y* lengths was fixed. Monolayer systems were prepared from the bilayer systems. Namely, the bilayer leaflets were separated into monolayers and put on the opposite sides of a water slab, and the *z*-dimension of the simulation box was enlarged to accommodate for the vacuum (gas phase). The box area in the *xy* plane was fixed to the value obtained for the equilibrated bilayer. In order to reduce the computational load, the number of water molecules was reduced to 12,000. The systems were equilibrated in canonical ensemble. All calculations were performed with periodic boundary conditions imposed on the system in all three directions. Particle mesh Ewald summation [62] was adopted to compute long-range electrostatic energies. The cutoff value of 12 Å was applied for Lennard-Jones potential. The anionic phospholipid membranes were neutralized by sodium cations [63]. All molecular dynamics simulations were performed using NAMD package [64]. The temperature and pressure were controlled using Langevin thermostat (LangevinDamping = 5) and barostat (LangevinPistonperiod = 200, LangevinPistonDecay = 100).

The initial structures of zalcitabine (ddC), stavudine (d4T), didanosine (ddI), and apricitabine (ATC) were built based on IUPAC names with the use of ChemDraw package from PerkinElmer Informatics. The absolute configuration of each molecule was verified. All structures were initially optimized using MM2 [65,66] force-field implemented in ChemDraw. Next, geometrical structures of all molecules were reoptimized at the DFT/B3LYP/6-311G(d,p) level of theory. All-atom CHARMM force-field for small molecules (cgenff) [67] was applied for the drug molecules. Missing parameters were derived using force-field toolkit [68] available in VMD package.

Numerous reports related to the POPC bilayer can be found in the literature [69,70,71]. These are both experimental and theoretical works. In contrast, the POPG bilayer has scarcely been explored. To the best of our knowledge, there are only a few experimental papers addressing the properties of pure POPG bilayers [72,73]. The experimental and theoretical areas per lipid in the POPC bilayer are similarly scattered. They vary from 63 Å^2^ to 68.3 Å^2^ [74,75,76,77,78]. The theoretical APL values are shifted towards larger APL values as compared with experimental data. The average APL from our simulation is 67.3 Å^2^ and is between the biggest experimental and theoretical values (66.6 Å^2^ and 68.3 Å^2^). MD simulations were performed at *T* = 300, 303, and 310 K. The experiments were conducted at 275, 297, 303, 310, and 315 K. The experimental APL value for POPG is 66 Å^2^ at 298 K. In our simulations performed at 310 K, we obtained a value of 69.4 Å^2^, which is reasonable with respect to surface variations for the zwitterionic POPC caused by the temperature increase. It was shown recently [79] that such a difference is related to the long-range dispersion contribution. Taking the dispersion into account brings experimental and theoretical values significantly closer together. Nevertheless, the availability of experimental data on ionic POPG bilayers is limited and further investigations are needed to find out how APL depends on the bilayer formation and the physicochemical conditions of the experiment.

The final drug–membrane systems were built by placing 18 drug molecules in water slabs of the systems containing previously equilibrated POPC/POPG monolayer/bilayer models. Nine molecules were put above and below each bilayer on 3 × 3 grid with random orientation of each molecule. The same strategy was applied in monolayer systems: 9 molecules were put above lower monolayer and 9 molecules were put under the upper monolayer. The molecules were initially immersed in water. In this way, 16 model systems were obtained. The box size in *xy* plane was fixed to the average values from the preliminary bilayer simulations. Monolayer systems were simulated in canonical ensemble. In the case of bilayer systems, (*N*,*A*,*p*_n_,*T*) ensemble was employed, where *N*, *A*, *p*_n_, and *T* correspond to number of particles, surface area, pressure normal to the surface, and temperature, respectively. The same surface area was used in monolayer and bilayer simulations independently of drug molecule. Physiological temperature was used in all simulations. The calculations were run for 120 ns. Trajectories were saved every 50 ps. The last 20 ns were considered as production run. The evolution of potential energy during the production run is shown in Figure S1 of Supplementary Materials to illustrate the achievement of equilibration. VMD [80] was used for trajectory visualization and analysis (radial pair distribution functions, HB counts, force-field energies). To calculate the density profiles, the programs developed by the authors were used.

Electronic structure calculations were performed at the DFT/B3LYP/6-311G(d,p) level of theory. In order to calculate chemical potential, hardness, and FF indices, optimized geometry of neutral drug molecules was frozen, and energy calculations were performed for cations and anions. ChelpG electrostatic-fitting scheme [81] was used to calculate atomic charges. Drug–lipid interactions were calculated at the AM1 level of theory. The structures were prepared via extraction from MD simulation frames. Coordinates of NRTIs together with all lipid molecules within a 3 Å distance were saved. AM1 interaction energies were calculated using the supermolecular approach. All QM calculations were performed in Gaussian09 suite.

## 4. Conclusions

The behavior of four antiretroviral drugs, namely zalcitabine, stavudine, didanosine, and apricitabine, in model mono- and bilayer systems was analyzed using molecular dynamics simulations. These drugs are used in human immunodeficiency virus (HIV) therapy. They belong to the same pharmacological group of nucleoside reverse-transcriptase inhibitors. According to the Biopharmaceutical Classification System (a tool used for classifying medicines based on dissolution, water solubility, and intestinal permeability), stavudine is an example of a Class I drug (high solubility in water and high permeability through intestinal barriers). The remaining three drugs belong to Class III of drugs (high solubility and low permeability).

The simulation models included bilayers and monolayers. Two phospholipids differing in polar headgroups, namely POPC and POPG, were tested. Antiretroviral drugs have been shown to have a greater affinity for POPG than POPC membranes. It was demonstrated that long-range electrostatic interactions are responsible for this effect. The predictions for the monolayer were consistent with those for the bilayers. This is important information from an experimental point of view. Taking into account that the composition and morphology of a monolayer can be easily controlled, the monolayer experiment becomes an important tool for understanding various phenomena in biological membranes.

It was demonstrated that drug molecules accumulate in phospholipid polar headgroups. Two adsorption modes were distinguished: adsorption to the phospholipid headgroups from the bottom with drug molecules immersed in water and adsorption into the headgroup region. The former is more dynamic in nature, and drug molecules can be pushed back to the water phase. The latter is more static and has a longer lifetime. The two modes differed in the degree of penetration of hydrophilic headgroups. Hydrogen bonds between drug molecules and phospholipid heads were responsible for both types of adsorption. It was shown that apricitabine penetrated the hydrophilic part of the POPC and POPG membranes more effectively than the other drugs. Van der Waals interactions between the S atoms and lipids were identified as the driving force for this effect. This observation was confirmed by calculating donor/acceptor properties on the grounds of conceptual DFT. High penetration of phospholipid membranes was also observed for stavudine. The electronic structure parameters of these two molecules, namely chemical potentials, distinguished them from the other molecules, confirming higher affinity towards the anionic membranes.

## Figures and Tables

**Table 1:** Bilayer/monolayer thickness parameters $d_{P-P}$/$d_{P-CT}$ and $d_{C2-C2}$/$d_{C2-CT}$. All values are in angstroms.

Bilayer	ddC	d4T	ddl	ATC
POPC: $d_{P-P}$	37.2 ± 0.2	37.3 ± 0.2	37.3 ± 0.2	37.4 ± 0.2
POPC: $d_{C2-C2}$	31.1 ± 0.1	31.2 ± 0.2	31.1 ± 0.2	31.3 ± 0.2
POPG: $d_{P-P}$	36.0 ± 0.2	36.1 ± 0.2	35.9 ± 0.3	36.2 ± 0.2
POPG: $d_{C2-C2}$	29.4 ± 0.5	30.0 ± 0.3	29.2 ± 0.5	30.4 ± 0.2
**Monolayer**				
POPC: $d_{P-CT}$	17.0 ± 0.3	17.0 ± 0.2	17.0 ± 0.2	17.2 ± 0.2
POPC: $d_{C2-CT}$	13.9 ± 0.3	13.9 ± 0.2	13.9 ± 0.2	14.1 ± 0.2
POPG: $d_{P-CT}$	16.5 ± 0.2	16.4 ± 0.2	16.4 ± 0.2	16.6 ± 0.2
POPG: $d_{C2-CT}$	13.6 ± 0.2	13.6 ± 0.2	13.6 ± 0.3	13.7 ± 0.2

**Table 2:** Number of hydrogen bonds formed by drug molecules with lipids and water molecules for monolayer and bilayer models. Standard geometry criterion was applied (donor–acceptor distance below 3 Å and angle cutoff 20°). The first line in each “System” entry gives the total number of HBs. The decomposition of this value into number of HBs with drug molecule acting as hydrogen donor/acceptor is shown in second/third lines.

	Monolayer		Bilayer	
System	HB with Lipid	HB with Water	HB with Lipid	HB with Water
ddC/POPC	1.57 ± 1.22 1.48 ± 1.19 0.09 ± 0.30	27.35 ± 4.93 7.41 ± 2.47 19.94 ± 3.94	0.21 ± 0.47 0.18 ± 0.43 0.03 ± 0.20	28.93 ± 5.10 7.29 ± 2.54 21.64 ± 4.28
d4T/POPC	2.65 ± 1.53 2.46 ± 1.29 0.19 ± 0.42	29.87 ± 5.11 8.56 ± 2.57 21.31 ± 4.30	0.87 ± 0.94 0.79 ± 0.88 0.09 ± 0.30	31.38 ± 4.87 8.71 ± 2.46 22.67 ± 4.07
ddI/POPC	1.13 ± 0.97 0.99 ± 0.88 0.14 ± 0.26	34.77 ± 5.25 8.59 ± 2.45 26.18 ± 4.62	0.64 ± 0.80 0.61 ± 0.78 0.03 ± 0.18	34.86 ± 5.44 8.63 ± 2.47 26.23 ± 4.64
ATC/POPC	4.79 ± 2.11 4.62 ± 2.24 0.17 ± 0.25	31.13 ± 4.84 12.11 ± 3.14 19.02 ± 3.85	6.07 ± 2.12 5.98 ± 2.09 0.09 ± 0.27	28.76 ± 4.76 11.42 ± 2.97 17.34 ± 3.72
ddC/POPG	1.30 ± 1.16 0.69 ± 0.77 0.61 ± 0.73	27.68 ± 4.78 6.23 ± 2.26 21.45 ± 4.14	1.44 ± 1.19 1.20 ± 1.07 0.24 ± 0.50	25.48 ± 4.55 5.93 ± 2.09 19.55 ± 3.95
d4T/POPG	3.39 ± 1.68 3.09 ± 1.90 0.30 ± 0.62	24.99 ± 4.67 6.86 ± 2.36 18.13 ± 4.31	5.89 ± 2.10 5.49 ± 1.96 0.41 ± 0.62	21.26 ± 4.73 5.95 ± 2.20 15.31 ± 3.58
ddI/POPG	1.87 ± 1.23 1.66 ± 1.21 0.21 ± 0.31	26.58 ± 4.94 6.52 ± 2.21 20.06 ± 3.97	1.20 ± 1.10 1.12 ± 1.04 0.08 ± 0.30	28.66 ± 5.17 7.30 ± 2.50 21.36 ± 4.63
ATC/POPG	7.80 ± 2.56 6.93 ± 2.25 0.87 ± 0.58	17.59 ± 4.02 7.46 ± 2.81 10.13 ± 2.97	7.07 ± 2.62 6.84 ± 2.56 0.23 ± 0.49	20.33 ± 4.15 10.22 ± 2.88 10.11 ± 2.96

**Table 3:** Chemical potential and hardness data computed at B3LYP/6-311G(d,p) level of theory. Polarizable continuum model was used to evaluate the environmental effect. All values are in electronvolts.

	Vacuum		Water	
Molecule	μ [eV]	η [eV]	μ [eV]	η [eV]
ddC	−3.6	9.0	−3.8	5.4
d4T	−3.9	9.1	−3.9	5.4
ddI	−3.5	9.0	−3.8	5.3
ATC	−3.9	8.7	−3.9	5.1

**Table 4:** Adsorption energies (interaction energies) of drug molecules in bilayer and monolayer systems. Energies (kcal/mol) were computed at the AM1 semiempirical level of theory. The structures were cut from MD trajectories (drug molecules and lipids within 3 Å from the drug).

Bilayer	ddC	d4T	ddl	ATC
POPC	−2.0 ± 0.7	−2.2 ± 0.6	−1.5 ± 0.7	−3.2 ± 1.2
POPG	−2.6 ± 1.4	−3.8 ± 1.9	−2.5 ± 1.4	−4.9 ± 1.7
**Monolayer**				
POPC	−2.5 ± 1.0	−2.7 ± 0.7	−3.3 ± 0.7	−2.9 ± 1.1
POPG	−2.4 ± 1.4	−3.4 ± 1.6	−1.3 ± 0.5	−4.5 ± 1.3

## Data Availability

Results are not deposited on publicly available servers, but may be available upon request.

## Supplementary Materials

The following supporting information can be downloaded at: https://www.mdpi.com/article/10.3390/molecules28176273/s1, Table S1: Interaction energies between NRTIs and lipids or water obtained from MD simulations of systems with POPC bilayer; Table S2: Interaction energies between NRTIs and lipids or water obtained from MD simulations of systems with POPG bilayer; Figure S1: Time dependence of total potential energy (V) during production MD runs.
