# Supplementary material for: Nucleoside Analog Reverse-Transcriptase Inhibitors in Membrane Environment: Molecular Dynamics Simulations
Source: Molecules. 2023 Aug 27;28(17):6273. doi: 10.3390/molecules28176273 (PMC10488468; doi:10.3390/molecules28176273)
Supplement: Supplementary file 1 [file molecules-28-06273-s001.zip › molecules-2552134-supplementary.pdf]

Supplementary Materials for

Nucleoside analog reverse-transcriptase inhibitors in mem-brane environment – molecular dynamic simulations

Anna Stachowicz-Kuśnierz, Beata Korchowiec and Jacek Korchowiec*

Faculty of Chemistry, Jagiellonian University, Gronostajowa 2, 30-387 Krakow, Poland

Table S1. Interaction energies between NRTIs and lipids or water obtained from MD simulations of systems with POPC bilayer. For each NRTI the first entry gives the energy obtained for the whole molecule, and the second entry gives the value per atom. All values are in kcal/mol.

System		Drug – lipids interaction energies			Drug – water interaction energies		
		Electrostatic	VDW	Total	Electrostatic	VDW	Total
Adsorbed molecules	ATC	-28.9	-22.1	-51.0	-30.7	-3.4	-34.2
		-1.1	-0.8	-2.0	-1.2	-0.1	-1.3
	d4T	-13.9	-19.7	-33.6	-30.2	-6.9	-37.1
		-0.5	-0.7	-1.2	-1.1	-0.2	-1.3
	ddC	-24.8	-12.0	-36.8	-40.9	-10.0	-50.9
		-0.9	-0.4	-1.3	-1.5	-0.4	-1.8
	ddI	-21.8	-18.8	-40.6	-35.1	-7.1	-42.2
		-0.8	-0.6	-1.4	-1.2	-0.2	-1.5
Molecules in solution	ATC	-1.0	-0.5	-1.5	-67.7	-15.8	-83.5
		0.0	0.0	-0.1	-2.6	-0.6	-3.2
	d4T	-0.4	-0.3	-0.6	-52.1	-17.7	-69.8
		0.0	0.0	0.0	-1.9	-0.6	-2.5
	ddC	-1.5	-0.5	-2.0	-67.5	-15.8	-83.3
		-0.1	0.0	-0.1	-2.4	-0.6	-3.0
	ddI	-0.8	-0.5	-1.3	-63.8	-16.8	-80.6
		0.0	0.0	0.0	-2.2	-0.6	-2.8

Table S2. Interaction energies between NRTIs and lipids or water obtained from MD simulations of systems with POPG bilayer. For each NRTI the first entry gives the energy obtained for the whole molecule, and the second entry gives the value per atom. All values are in kcal/mol.

System		Drug – membrane interaction energies			Drug – water interaction energies		
		Electrostatic	VDW	Total	Electrostatic	VDW	Total
Adsorbed molecules	ATC	-19.0	-20.8	-39.8	-11.7	-5.9	-17.6
		-0.7	-0.8	-1.5	-0.4	-0.2	-0.7
	d4T	-8.2	-19.8	-28.0	-11.4	-7.0	-18.3
		-0.3	-0.7	-1.0	-0.4	-0.2	-0.7
	ddC	-25.2	-13.2	-38.5	-31.9	-9.2	-41.2
		-0.9	-0.5	-1.4	-1.1	-0.3	-1.5
	ddI	-8.9	-13.5	-22.3	-23.7	-10.7	-34.4
		-0.3	-0.5	-0.8	-0.8	-0.4	-1.2
Molecules in solution	ATC	-7.7	-0.4	-8.1	-58.3	-15.9	-74.2
		-0.3	0.0	-0.3	-2.2	-0.6	-2.9
	d4T	-3.0	-0.3	-3.4	-47.2	-17.6	-64.8
		-0.1	0.0	-0.1	-1.7	-0.6	-2.3
	ddC	-9.5	-0.5	-10.0	-60.3	-16.1	-76.3
		-0.3	0.0	-0.4	-2.2	-0.6	-2.7
	ddI	-3.2	-0.5	-3.7	-55.8	-17.4	-73.1
		-0.1	0.0	-0.1	-1.9	-0.6	-2.5

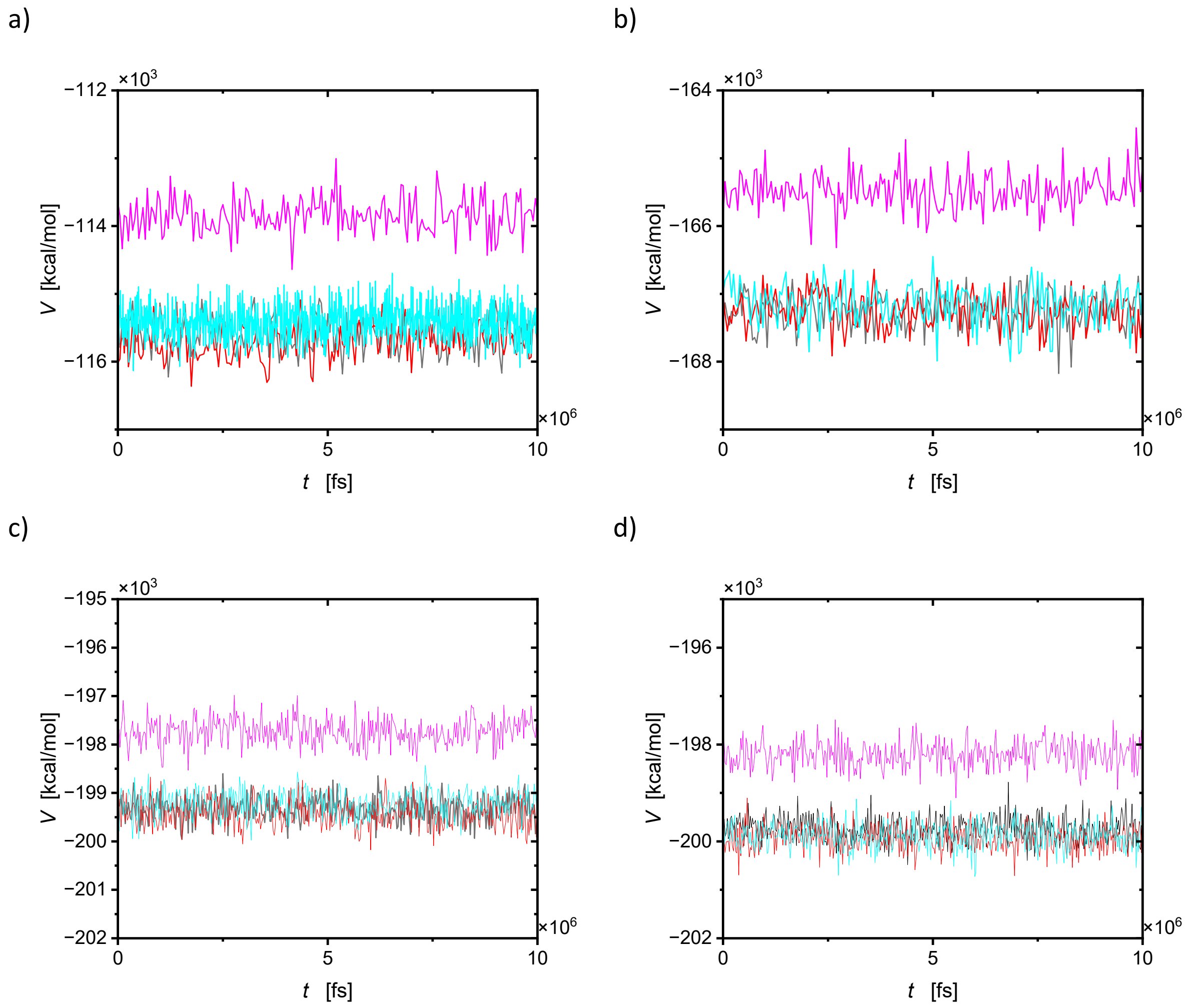

Figure S1

Time dependence of total potential energy (V) during production MD runs. Color code: black - ATC; magenta – DDI; red - d4T; cyan - ddC. Panels a,b , c and d correspond to POPC monolayer, POPC bilayer, POPG monolayer, and POPG bilayer respectively.
